# Bridging the Gap Between Aging and Disability Research

**DOI:** 10.1093/geroni/igaf122.976

**Published:** 2025-12-31

**Authors:** Michelle Putnam

**Affiliations:** University of Massachusetts Boston, Boston, Massachusetts, United States

## Abstract

Efforts to bridge aging and disability research date back over forty years. Increased longevity of persons experiencing early and mid-life disability has prompted bridging work, but conceptual and methodological differences reflecting disciplinary-specific approaches to age-based disability-related research have research restricted synergy across fields. This presentation describes the evolution of efforts to bridge aging and disability research over time, historical and current variances in how aging and disability research has approached the study of disability, and what resultant challenges for aligning and bridging aging and disability research are. Issues identified include use of medical vs social models of disability, operationalization and measurement of disability, and interpretation of disability-related data. Legal, social and historical factors that have shaped approaches to investigating aging and disability are also discussed. Recommendations regarding how to address long-standing gaps in knowledge and barriers to bridging work are made.

